# Cervical Metastasis From Primary Breast Carcinoma: A Case Report and Review of Extragenital Tumors Involving the Uterine Cervix

**DOI:** 10.1002/ccr3.72993

**Published:** 2026-06-22

**Authors:** Mario Assenza, Adele Zappalà, Armando Nallbani, Alessandra Baldoni, Jacopo Wabersich

**Affiliations:** ^1^ Dipartimento di Salute Della Donna e del Bambino Università Degli Studi di Padova Padova Italy; ^2^ Unità di Ginecologia e Ostetricia, ULSS 3 “Serenissima” Mirano‐Dolo Italy; ^3^ UOC di Oncologia Medica, ULSS 3 “Serenissima” Mirano Italy

**Keywords:** breast cancer metastasis, cancer recurrence, cervical metastasis, ductal carcinoma, immunohistochemistry, uterine metastasis

## Abstract

Cervical metastases from breast carcinoma should be considered in the differential diagnosis of uterine lesions in patients with prior breast cancer. Re‐biopsy is essential to detect receptor discordance (ER/PR up to 29%) and guide therapy. GCDFP‐15, mammaglobin, and GATA3 confirm mammary origin.

## 1 Introduction

Breast cancer is the most common female malignancy, accounting for 55,900 new cases in Italy in 2024 [1]. Histologically, invasive forms include invasive ductal carcinoma (IDC, ~75%), invasive lobular carcinoma (ILC, 10%–15%), and minor subtypes such as tubular, mucinous, and medullary carcinoma (< 2%–6% each). Tumor grading is assigned according to the Nottingham Histologic Score, which stratifies tumors into G1 (well differentiated, ~20%), G2 (~35%), and G3 (~46%). The receptor profile: estrogen receptor (ER), progesterone receptor (PR), HER2, and Ki‐67, defines molecular subtypes (luminal A/B, HER2‐positive, basal‐like) and guides therapeutic decision‐making.

Metastases to the female genital organs are uncommon: according to Piura et al. [2], the ovaries are involved in 13.2%–37.8% of cases and the uterus in approximately 10%. ILC shows a greater propensity for genital tract metastasis compared to IDC, attributed to loss of E‐cadherin expression (via *CDH1* gene inactivation), which disrupts cell‐to‐cell adhesion and promotes a diffuse infiltrative pattern [3]. Endometrial metastases typically present with abnormal uterine bleeding [4]. A diagnosis of certain mammary origin requires: (a) histopathological concordance with the primary tumor and exclusion of synchronous uterine neoplasms; (b) absence of correlation with hormonal therapy (e.g., tamoxifen); and (c) positivity for breast‐specific immunohistochemical markers (GCDFP‐15, mammaglobin) [5].

## 2 Case History

A 59‐year‐old woman (born 1965) presented to our Gynecology and Obstetrics Unit in January 2024. Her medical history was significant for invasive ductal carcinoma G2, stage pT1cN2a, ER 89%, PR 80%, Ki‐67 30%, HER2 0, diagnosed in 2015. She had undergone wide local excision (right quadrantectomy) with axillary lymph node dissection, followed by adjuvant therapy consisting of four cycles of doxorubicin and cyclophosphamide, weekly intravenous paclitaxel 80 mg/m^2^ for 12 cycles, complementary radiotherapy, and endocrine therapy with an LH‐RH analogue plus letrozole 2.5 mg/day, completed in July 2020.

From June 2021, a progressive rise in the tumor marker CA 15‐3 was observed (reference range: 0–32.4 U/mL), reaching a stable plateau of approximately 125 U/mL by April 2022. The patient was referred for evaluation of atypical vaginal bleeding. PET‐CT performed in May 2023 revealed an area of heterogeneous and irregular hypermetabolism within the uterus (SUVmax 7.39). Transvaginal ultrasound demonstrated mild endometrial thickening of 5.2 mm (Figure 1). [18F]FDG PET/CT was crucial for disease characterization, identifying the hypermetabolic uterine lesion and guiding diagnosis and staging. Beyond its established role in baseline staging, it is increasingly used to monitor response to immunotherapy. However, phenomena such as pseudoprogression and immune‐related inflammation can complicate interpretation. PET/CT provides metabolic information that may help distinguish immune activity from true progression, although this remains challenging. In hematologic settings (e.g., CAR‐T therapy), it has demonstrated high accuracy in response assessment and detection of immune‐related adverse events. As immunotherapy use in solid tumors expands, standardized PET/CT response criteria (such as PECRIT and iRECIST‐integrated approaches) are expected to play an increasing role in patient management [6]. In January 2024, operative hysteroscopy with multiple biopsies of the endometrium and cervix was performed. Histological examination identified a poorly differentiated carcinoma consistent with apocrine carcinoma, with the following immunohistochemical (IHC) profile: ER−, PR−, Ki‐67 20%, HER2 negative, androgen receptor (AR) 90%, PD‐L1 5%. Restaging contrast‐enhanced CT of the chest and abdomen (March 2024) documented a 7‐mm lateral cervical lymph node, an 8 × 5‐mm marginal costal pleural thickening, a globular uterus with accentuation at the fundus, and several subcentimetric mesenteric lymph nodes. A central venous port was placed on 15 March 2024, and first‐line chemotherapy with nab‐paclitaxel (Days 1, 8, 15) and atezolizumab (Days 1, 15) was initiated on 20 March 2024. Treatment is ongoing and well tolerated both clinically and biochemically (Figure 2).

**FIGURE 1:**
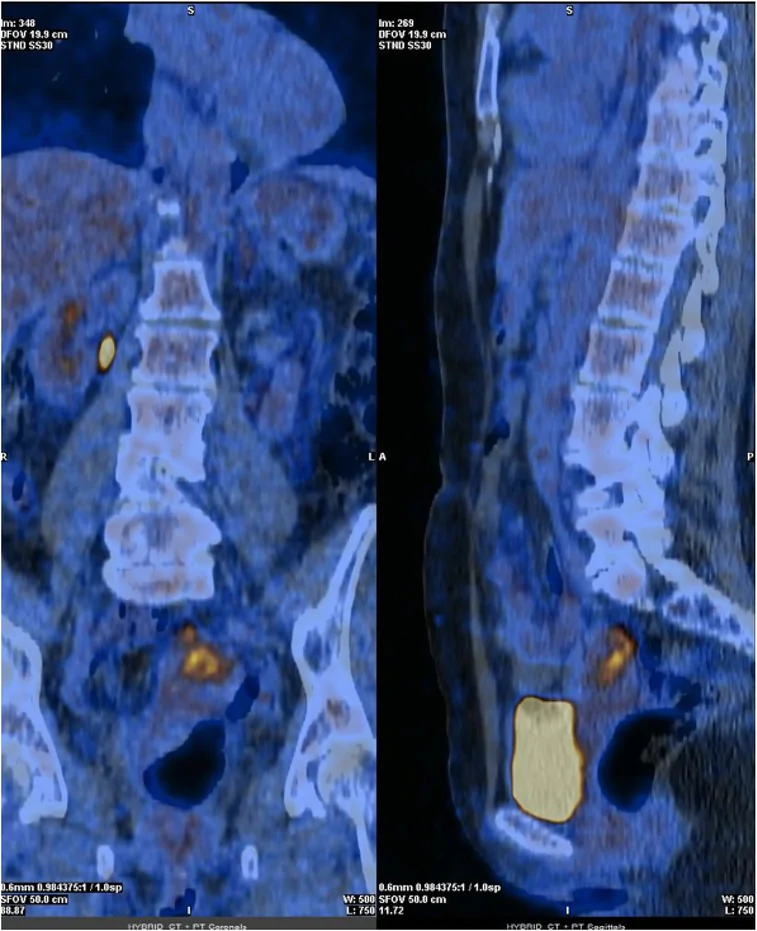
(A) Coronal projection of a PET‐CT scan showing hypermetabolic activity in the cervix (blue arrow) and uterus (red arrow), with a maximum SUV of 7.39. (B) Sagittal projection of the same PET‐CT scan showing the extent of uterine and cervical involvement, as well as pelvic lymph nodes (green arrows).

**FIGURE 2:**
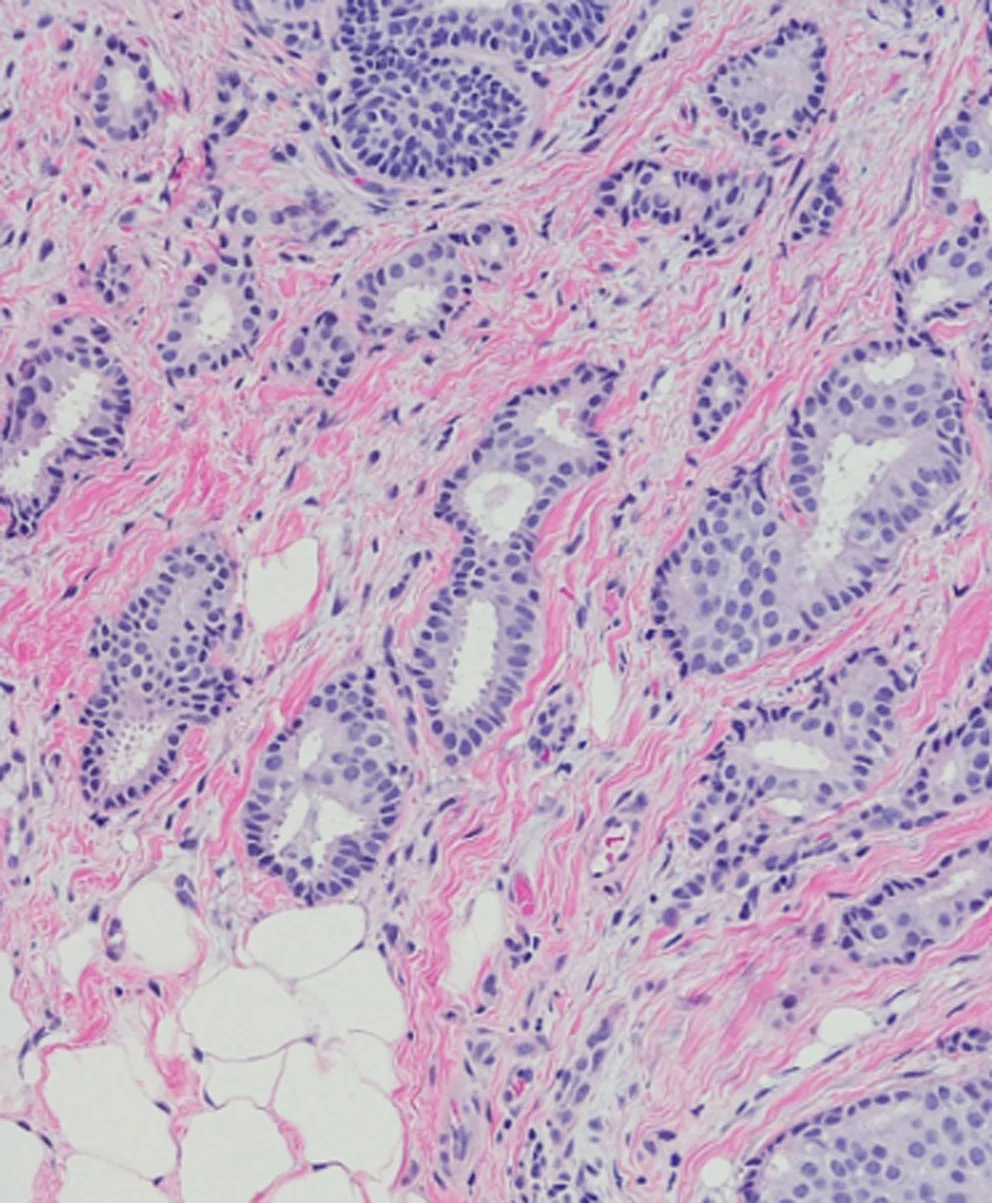
Histological slide of invasive ductal carcinoma of the breast.

## 3 Differential Diagnosis, Investigation and Treatment

To characterize the neoplasm and determine its origin, histological specimens from the 2015 and 2024 biopsies were reviewed by the same group of pathologists at the Angelo Hospital in Mestre. Specimens were classified according to the WHO Classification of Breast Tumors (2019), including all parameters required by the American Joint Committee on Cancer (AJCC) in its most recent edition. The pathological report was completed with assessment of prognostic factors: estrogen receptors (anti‐ER), progesterone receptors (anti‐PR), proliferative activity (anti‐Ki‐67), and HER2 oncogene overexpression.

The differential diagnosis included the following hypotheses:

- Synchronous primary endometrial carcinoma: excluded based on histological morphology and IHC profile (ER−, PR−, AR +90%), incompatible with the most common endometrial primary subtypes.
- Tamoxifen‐related endometrial neoplasia: excluded as the patient had never received tamoxifen
- Metastasis from primary breast carcinoma: confirmed by concordance of the IHC profile with the 2015 primary tumor, notably the positivity for AR and PD‐L1, and by the positivity for GCDFP‐15 and mammaglobin, highly specific markers of mammary origin [7].

In accordance with the American Society of Clinical Oncology/College of American Pathologists (ASCO/CAP) guidelines, which recommend re‐evaluation of receptor status in metastatic disease relative to the primary tumor, a complete IHC profile was performed on the 2024 biopsy. Results demonstrated significant receptor discordance compared to 2015 (ER− vs. ER 89%; PR− vs. PR 80%), guiding the therapeutic choice toward the combination of chemotherapy (nab‐paclitaxel) and immunotherapy (atezolizumab), given the PD‐L1 positivity.

The combination of nab‐paclitaxel and atezolizumab is supported by the IMpassion130 trial, which showed improved PFS in PD‐L1–positive (IC ≥ 1%) metastatic triple‐negative breast cancer, with benefit observed regardless of PD‐L1 expression level and no advantage in PD‐L1–negative patients. The 5% PD‐L1 positivity in our case meets the eligibility criteria, supporting the chosen treatment [8, 9].

The metastatic lesion's apocrine, AR‐positive (90%) profile is consistent with the luminal androgen receptor (LAR) subtype of TNBC, characterized by androgen‐driven signaling, frequent PIK3CA mutations, and relative chemoresistance [10]. Emerging data suggest potential sensitivity of this subtype to targeted strategies such as CDK4/6 and AKT inhibition. The strong AR expression, absent in the primary tumor, likely reflects clonal evolution and molecular plasticity during metastatic progression [11, 12].

## 4 Conclusions and Results

This case describes a rare cervicouterine metastasis from invasive ductal breast carcinoma, arising approximately 9 years after primary treatment, consistent with the documented tendency of breast cancer metastases to manifest years after the initial diagnosis [13].

The key diagnostic element was the IHC profile of the biopsied lesion, which showed positivity for GCDFP‐15 and mammaglobin, highly specific markers of mammary origin, together with a receptor pattern (ER−, PR−, AR 90%, PD‐L1 5%) incompatible with primary endometrial neoplasms and consistent with the known receptor discordance between primary tumor and metastases, estimated in the literature at 23.7% for ER and 28.9% for PR [14].

Treatment with nab‐paclitaxel and atezolizumab was initiated in March 2024 and was well tolerated clinically and biochemically. Follow‐up is ongoing; at the time of the last available assessment, no severe adverse events had been recorded and the safety profile of the regimen was acceptable. Definitive therapeutic response will be evaluated at the scheduled oncological restaging.

This case highlights three key clinical messages: (a) cervicouterine metastases from breast carcinoma, though rare, should be considered in the differential diagnosis of uterine lesions in patients with a history of breast cancer, even many years after primary treatment; (b) biopsy of the metastatic lesion is essential for updated receptor characterization, which may differ substantially from the primary tumor and determine a different therapeutic approach; (c) annual gynecological follow‐up, including transvaginal ultrasound and cervical cytology, is recommended even in asymptomatic patients with a history of breast cancer, to enable early detection of secondary pelvic involvement. This case differs from previously reported breast‐to‐cervix metastases in several aspects [3, 15]. First, the primary tumor was invasive ductal carcinoma (IDC), whereas most cervical metastases arise from lobular carcinoma (ILC), reflecting the known role of E‐cadherin loss; the long nine‐year disease‐free interval further underscores its rarity. Second, the complete ER/PR loss at metastasis, compared to the strongly hormone receptor–positive primary tumor, represents an extreme form of receptor discordance (ER 23.7%, PR 28.9%) and led to a shift from endocrine therapy to chemo‐immunotherapy [14]. Third, the emergence of marked apocrine differentiation with strong AR expression (90%), absent in the primary tumor, suggests subclonal selection toward an androgen receptor–driven LAR‐like phenotype, a feature not previously described in cervical metastases from IDC.

## 5 Discussion

### 5.1 Epidemiology and Pathways of Dissemination

Secondary involvement of the uterine cervix by extragenital malignancies is exceedingly rare, accounting for a very small proportion of genital tract metastases (≤ 3.4% and < 2% in major series) [16, 17]. The most common primary sites include the gastrointestinal tract, followed by breast and urothelial carcinomas, with a mean age of approximately 55.5 years and variable latency depending on tumor type [13, 15].

This rarity is largely explained by cervical anatomical features such as small size, dense stroma, low vascularity, and centrifugal lymphatic drainage [13, 18]. Metastatic spread occurs mainly via hematogenous routes, but may also involve retrograde lymphatic dissemination or transperitoneal extension in advanced disease [13, 15].

At the molecular level, epithelial‐to‐mesenchymal transition (EMT) enables tumor cell dissemination, supported by cytoskeletal remodeling and mRNA translation dynamics [19, 20]. Metastasis involves complex molecular reprogramming, including epigenetic and transcriptomic changes, contributing to phenotypic divergence and receptor discordance. Advances in multi‐omics and liquid biopsy technologies are refining the understanding of clonal evolution and supporting more precise therapeutic strategies in metastatic breast cancer [21] (Figure 3).

**FIGURE 3:**
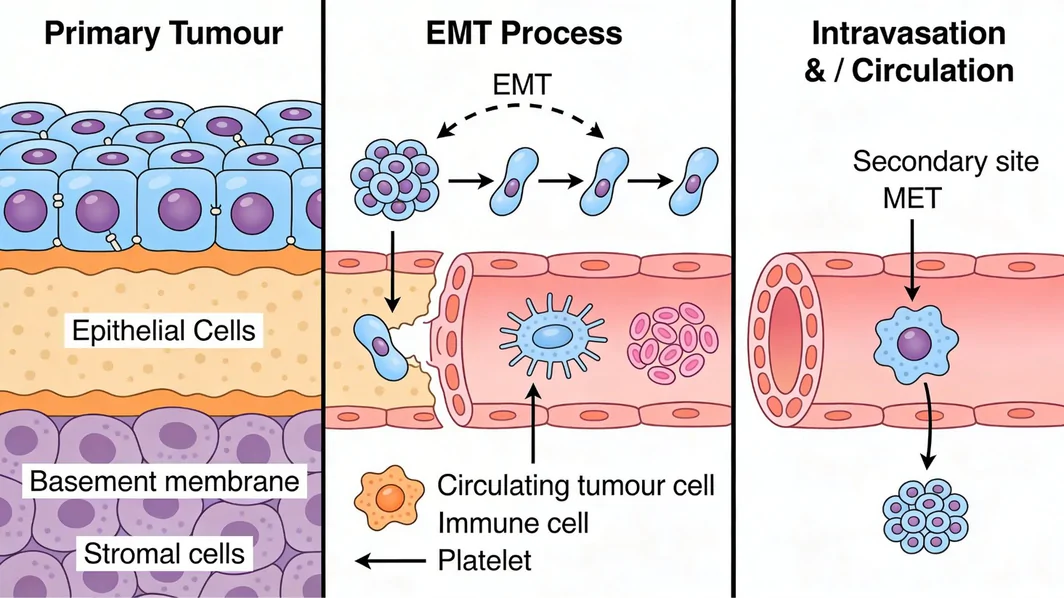
Epithelial‐to‐mesenchymal transition (EMT) is a process by which epithelial cells lose their cell polarity and cell‐to‐cell adhesion, acquiring migratory and invasive properties to become mesenchymal stem cells.

### 5.2 Breast Carcinoma (40 Reported Cases)

Breast carcinoma is the most common extragenital malignancy to metastasize to the uterine cervix, representing 42.9% of extragenital cervical metastases in some series [13]. To date, approximately 40 cases have been described [15]. Although IDC accounts for approximately 80% of all breast carcinomas, ILC exhibits a significantly greater propensity for female genital tract metastasis due to loss of E‐cadherin expression (CDH1 gene inactivation), which impairs intercellular cohesion and promotes a diffuse single‐file infiltrative pattern [3]. Lobular metastases are characterized histologically by discohesive cells infiltrating in single file, preserved surface epithelium, and absence of desmoplastic reaction, a pattern misdiagnosed as primary squamous cell carcinoma in 1 in 5 cases in the Turashvili series [15]. Ductal metastases, by contrast, tend to form nests or infiltrating glands, mimicking conventional endocervical adenocarcinoma [15]. The molecular variability observed in metastatic breast cancer, including shifts in hormone receptor status, HER2 expression, and subtype identity, is further contextualized by recent studies characterizing germline mutational profiles and their clinicopathological associations in triple‐negative breast cancer [22]. These findings underscore that molecular heterogeneity is not merely a byproduct of tumor evolution, but is partly shaped by germline‐level predispositions that influence the metastatic phenotype [23].

Patients may be asymptomatic (up to 32% in early stages) or present with abnormal vaginal bleeding, vaginal discharge, or, in advanced cases, pelvic symptoms indistinguishable from primary cervical carcinoma [24]. The interval between primary breast cancer diagnosis and cervical metastasis is typically 5–20 years, with cases documented beyond 20 years [25]. Median survival is approximately 28 months [15].

Representative recent cases include: Shan et al. [14], a 40‐year‐old with ER+/HER2− breast cancer who, after 7 years of endocrine therapy, was found on screening cytology to have HSIL with HPV positivity; cervical biopsy confirmed grade II ILC (E‐cadherin−, GATA3+, p16−), isolated cervical involvement on PET‐CT (SUVmax 4.9), treated with total hysterectomy plus bilateral salpingo‐oophorectomy followed by fulvestrant + abemaciclib (PFS 31 months); Dye et al. [25], IDC recurrence > 20 years after primary diagnosis presenting with post‐menopausal spotting, treated with palliative radiotherapy and chemotherapy; Boudagh et al. [26], bilateral IDC (stage IIIA–IIIB) with heavy vaginal bleeding as the manifestation of cervical metastasis in the setting of widely disseminated disease; Thouvenot et al. [27], IDC recurrence in an 86‐year‐old patient presenting with anemia and metrorrhagia.

### 5.3 Gastric Carcinoma (< 40 Reported Cases)

Gastric carcinoma accounts for 11.1% of extragenital cervical metastases [13]. In the seminal series by Imachi et al. (1993), the largest reported with 16 cases, 6 of 16 patients had the cervical metastasis discovered simultaneously with or prior to the primary gastric diagnosis [28]. The mean interval between primary diagnosis and cervical metastasis is approximately 35.8 months. The histological mimicry between diffuse‐type gastric adenocarcinoma and gastric‐type endocervical adenocarcinoma (an HPV‐independent neoplasm with mucinous columnar cells, CK7+, CK20+, HIK1083+, PAX8−) makes the differential diagnosis particularly challenging [15]. Bahall et al. [13] describe a case in which gastric adenocarcinoma was incidentally discovered on histological examination following hysterectomy for symptomatic leiomyomas; Matsushita et al. [29] report a case clinically simulating stage IIIB cervical carcinoma; Yamamoto et al. [30] document a late isolated cervical metastasis arising 10 years after curative gastrectomy, demonstrating that the latency period may be exceedingly prolonged.

### 5.4 Colorectal Carcinoma (~35 Reported Cases Until 2014)

Colorectal carcinoma (CRC) is the most numerically represented gastrointestinal subtype in cervical metastases; 17 cases were identified in the Turashvili series [15] and approximately 35 cases had been published through 2014 [31]. The mean interval from primary diagnosis to cervical metastasis is approximately 17 months [32]. The IHC profile (CK20+, CDX2+, SATB2+, CK7−) is essential to distinguish metastatic CRC from primary intestinal‐type endocervical adenocarcinoma; dirty endoluminal necrosis was present in 76.5% of CRC cases in the Turashvili series [15]. Sassi et al. [33] describe a 38‐year‐old patient with a 9 × 6 × 5‐cm cervical mass causing left hydronephrosis, treated with hemostatic decompressive radiotherapy (45 Gy) with complete local clinical response; Chereau et al. [32] report vaginal extension 3 years after the primary tumor, underscoring the role of IHC in diagnosis.

### 5.5 Renal Cell Carcinoma (< 10 Reported Cases)

Renal cell carcinoma (RCC) is notable for its unpredictable metastatic behavior and capacity for late metastases to unusual sites. The predominance of left‐sided primary tumors is explained by retrograde venous extension via the left renal vein to the left ovarian vein [28]. Bozaci et al. [28] describe the second‐youngest patient reported (19 years), with synchronous metastases to the cervix and vagina 1 year after radical nephrectomy; Seseke et al. [34] report an isolated cervical metastasis from clear‐cell RCC, one of fewer than 5 cases known at publication; Beriwal et al. (2009) describe a 45‐year‐old with RCC cervical metastasis arising 4 years after treatment of a dual primary (colon + RCC), managed with radical hysterectomy and sunitinib [34]; Sree et al. (2025) document synchronous vaginal, cervical, and pulmonary metastases from left‐sided RCC, treated with pazopanib 800 mg/day.

### 5.6 Lung Carcinoma

Only 3 cases of pulmonary carcinoma metastatic to the cervix have been described in the literature [15]. TTF‐1 and napsin‐A immunopositivity are diagnostic. Kai et al. [35] report a 69‐year‐old postmenopausal woman with a cauliflower‐like cervical mass with bilateral parametrial invasion, arising 4 years after video‐assisted thoracoscopic right upper lobectomy for adenocarcinoma (stage IIIb, pT3N1M0), with TTF‐1‐confirmed mammary‐type IHC.

### 5.7 Pancreatic Carcinoma

Hartsough et al. [36] describe what is believed to be the only reported case of isolated pancreatic ductal adenocarcinoma recurrence to the uterine cervix, highlighting the morphological overlap with gastric‐type endocervical adenocarcinoma as a potential diagnostic pitfall.

### 5.8 Gallbladder, Urothelial Carcinoma, and Cutaneous Melanoma

Gallbladder carcinoma cervical metastases (8 cases in the literature) characteristically present within less than 1 year of primary diagnosis, the shortest latency of all reported primary sites, with deep stromal invasion, infiltrating‐pagetoidal growth replacing both squamous and glandular epithelia, and prominent LVSI [15]. Urothelial carcinoma (fewer than 10 cases) shows a high risk of misdiagnosis: two of fivecases in the Turashvili series were initially interpreted as HSIL or adenocarcinoma in situ; GATA3, p63, and CK7 are the key diagnostic markers [15]. Cutaneous melanoma accounts for 9 cases; the latency is typically less than 1 year, and S100, HMB‐45, melan‐A, and SOX10 are diagnostic regardless of origin. Differential diagnosis with primary cervical melanoma (~80 cases worldwide) relies on the absence of junctional activity and the clinical history of a prior cutaneous primary [15].

### 5.9 Histological Patterns, Immunohistochemistry, and Differential Diagnosis

In the Turashvili series [15], cervical metastases from extragenital primaries most commonly showed an infiltrative growth pattern (63.8%), followed by mixed infiltrative–pagetoid (27.7%) and purely pagetoid patterns (6.4%), with frequent lymphovascular space invasion (68.3%) and deep stromal infiltration (> 50% in 70.2%). Notably, 12.8% of cases were initially misdiagnosed as primary cervical tumors, highlighting important diagnostic challenges. Receptor discordance between primary and metastatic lesions reflects clonal evolution and treatment‐related selective pressures; in triple‐negative breast cancer, germline alterations (e.g., BRCA1/2) further contribute to tumor heterogeneity and metastatic behavior. The marked receptor shift observed in the present case underscores the importance of repeat biopsy and receptor reassessment at recurrence, in line with ASCO/CAP recommendations.

Histologically, features favoring metastatic disease include stromal infiltration with sparing of surface epithelium, deep invasion without intraepithelial lesions, discohesive single‐file cells, absence of desmoplasia, prominent LVSI, and negativity for Müllerian markers such as PAX8 and p16, alongside positivity for non‐Müllerian markers. In contrast, expression of Müllerian markers (PAX8, WT1) and diffuse p16 positivity support a primary cervical origin [15] (Table 1).

**TABLE 1:** Summary of reported cases in the literature by primary tumor origin.

Primary site	Reported cases	Interval from primary	Main symptom	Main mechanism	Key IHC
Breast (ILC/IDC)	~40 cases	5–20 years	Vaginal bleeding/asymptomatic	Hematogenous	GATA3+, ER/PR+, E‐cad− ILC
Stomach	< 40 cases	35.8 months (mean)	Vaginal bleeding/incidental	Retrograde lymphatic	CK7+, CDX2+, PAX8−
Colorectum	~35 cases	17 months (mean)	Vaginal bleeding	Hematogenous/lymphatic	CK20+, CDX2+, SATB2+, CK7−
Kidney (RCC)	< 10 cases	Variable (late onset)	Post‐menopausal bleeding	Retrograde venous	CD10+, PAX8+, RCC+
Lung	Three cases	Variable	Vaginal bleeding/mass	Hematogenous	TTF‐1+, napsin‐A+
Pancreas	1–2 cases	Variable	Not specified	Hematogenous/lymphatic	CK7+, CA19‐9+, PAX8−
Gallbladder	8 cases	< 1 year	Cervical mass	Lymphatic	CK7+, CK20±, PAX8−
Cutaneous melanoma	Nine cases	< 1 year	Vaginal bleeding/mass	Hematogenous	S100+, HMB‐45+, SOX10+
Urothelial carcinoma	< 10 cases	3 years (mean)	Vaginal bleeding/incidental	Lymphatic/hematogenous	CK7+, GATA3+, p63+, CK20 var.

Abbreviations: E‐cad, E‐cadherin; IDC, invasive ductal carcinoma; IHC, immunohistochemistry; ILC, invasive lobular carcinoma; RCC, renal cell carcinoma; var., variable.

### 5.10 Treatment and Prognosis

No standardized treatment guidelines exist for cervical metastases from extragenital primaries; management is individualized within a multidisciplinary setting [13, 33]. Surgery may be considered for isolated, resectable lesions, particularly in breast cancer [13], while systemic therapy is the main approach in multifocal disease, tailored to tumor type (e.g., fluoropyrimidine–platinum for gastric cancer; CDK4/6 inhibitors plus endocrine therapy for ER‐positive breast cancer) [13, 14]. Palliative radiotherapy is effective for symptom control [33]. Prognosis is generally poor, with median survival of approximately 4 months for gastric and 28 months for breast metastases, while better outcomes are seen in isolated colorectal cases amenable to radical treatment [15, 33]. (Table 2).

**TABLE 2:** Characteristic immunohistochemical profiles by primary tumor site.

Primary site	Characteristic IHC profile
Ductal breast carcinoma	CK7+, GATA3+, mammaglobin+, ER/PR+, HER2 variable, E‐cadherin+
Lobular breast carcinoma	CK7+, GATA3+, ER/PR+, E‐cadherin−, p120 cytoplasmic+
Gastric carcinoma	CK7±, CK20±, CDX2+, PAX8−, HIK1083+
Colorectal carcinoma	CK20+, CDX2+, SATB2+, CK7−
Lung adenocarcinoma	TTF‐1+, napsin‐A+, CK7+
Renal cell carcinoma	CD10+, RCC antigen+, PAX8+, CK7−
Urothelial carcinoma	CK7+, GATA3+, p63+, CK20 variable
Cutaneous melanoma	S100+, HMB‐45+, melan‐A+, SOX10+, AE1/AE3−

At progression, reassessment of tumor biology is essential. Re‐evaluation for HER2‐low status is recommended, as many tumors initially classified as HER2‐negative may be reclassified. Trastuzumab deruxtecan has demonstrated significant survival benefit in HER2‐low metastatic breast cancer, while sacituzumab govitecan is an effective option in both HR‐positive/HER2‐negative and triple‐negative disease [37]. Their role in AR‐positive, apocrine‐type TNBC remains to be defined [38].

Across tumor types, breast metastases show longer latency and better outcomes; gastric metastases have the worst prognosis, and colorectal metastases may be curable when isolated [15, 33]. Overall, optimal management relies on multidisciplinary care integrating updated histopathological assessment, biomarker‐driven systemic therapy, and selective local treatment strategies.

## Author Contributions

**Adele Zappalà:** conceptualization, investigation, writing – original draft, methodology, validation, writing – review and editing. **Armando Nallbani:** data curation, supervision, resources, project administration, formal analysis, conceptualization, investigation, methodology. **Jacopo Wabersich:** resources, supervision, data curation, project administration. **Mario Assenza:** conceptualization, investigation, writing – original draft, methodology, validation, writing – review and editing. **Alessandra Baldoni:** conceptualization, investigation, writing – original draft, methodology, validation, writing – review and editing.

## Funding

The authors have nothing to report.

## Ethics Statement

The authors have nothing to report.

## Consent

Written informed consent was obtained from the patient for publication of clinical data and images.

## Conflicts of Interest

The authors declare no conflicts of interest.

## Supporting information


**Data S1:** Additional Information

## Data Availability

All data are available for scientific purposes if needed.
