# Supplementary material for: Cervical Metastasis From Primary Breast Carcinoma: A Case Report and Review of Extragenital Tumors Involving the Uterine Cervix
Source: Clin Case Rep. 2026 Jun 22;14(7):e72993. doi: 10.1002/ccr3.72993 (PMC13286867; doi:10.1002/ccr3.72993)
Supplement: Supplementary file 1 — Data S1: Additional Information [file CCR3-14-e72993-s001.docx]

**Additional Information**

**Disclosures**

**Human subjects:** Consent was obtained or waived by all participants in this study. Written informed consent was obtained from the patient for publication of clinical data and images. This case report was conducted in accordance with institutional guidelines and ethical standards. No formal ethics approval was required for this retrospective case report.

**Conflicts of interest**: all authors declare the following: Payment/services info: All authors have declared that no financial support was received from any organization for the submitted work. Financial relationships: All authors have declared that they have no financial relationships at present or within the previous three years with any organizations that might have an interest in the submitted work. Other relationships: All authors have declared that there are no other relationships or activities that could appear to have influenced the submitted work.

**Author Contributions**

**Concept and design**: Mario Assenza, Adele Zappalà

**Acquisition, analysis, or interpretation of data:** Mario Assenza, Adele Zappalà

**Drafting of the manuscript:** Mario Assenza, Adele Zappalà

**Critical review of the manuscript for important intellectual content:** Mario Assenza, Adele Zappalà, Armando Nallbani, Jacopo Wabersich

**Supervision**: Armando Nallbani, Jacopo Wabersich
